# Sonographic Evaluation of Gastric Residual Volume during Enteral Nutrition in Critically Ill Patients Using a Miniaturized Ultrasound Device

**DOI:** 10.3390/jcm10214859

**Published:** 2021-10-22

**Authors:** Tizian Jahreis, Jessica Kretschmann, Nick Weidner, Thomas Volk, Andreas Meiser, Heinrich Volker Groesdonk

**Affiliations:** 1 Department of Anaesthesiology, Intensive Care and Pain Medicine, Saarland University Medical Center and Saarland University Faculty of Medicine, 66421 Homburg, Germany; tizian.jahreis@outlook.com (T.J.); jessica.kretschmann@uks.eu (J.K.); thomas.volk@uks.eu (T.V.); andreas.meiser@uks.eu (A.M.); 2 Department of Interdisciplinary Critical Care Medicine and Intermediate Care, Helios Klinikum Erfurt, 99089 Erfurt, Germany; nick.weidner@helios-gesundheit.de

**Keywords:** intensive care, gastric ultrasound, multi-organ point-of-care ultrasound (MOPOCUS), critical illness, enteral nutrition

## Abstract

Background: To assess the risk of aspiration, nutrient tolerance, and gastric emptying of patients in ICUs, gastric ultrasound can provide information about the gastric contents. Using established formulas, the gastric residual volume (GRV) can be calculated in a standardized way by measuring the gastric antrum. The purpose of this study was to determine the GRV in a cohort of enterally fed patients using a miniaturized ultrasound device to achieve knowledge about feasibility and the GRV over time during the ICU stay. The findings could contribute to the optimization of enteral nutrition (EN) therapy. Methods: A total of 217 ultrasound examinations with 3 measurements each (651 measurements in total) were performed twice daily (morning and evening) in a longitudinal observational study on 18 patients with EN in the interdisciplinary surgical ICU of Saarland University Medical Center. The measured values of the GRV were analyzed in relation to the clinical course, the nutrition, and other parameters. Results: Measurements could be performed without interrupting the flow of clinical care and without pausing EN. The GRV was significantly larger with sparsely auscultated bowel sounds than with normal and excited bowel sounds (*p* < 0.01). Furthermore, a significantly larger GRV was present when using a high-caloric/low-protein nutritional product compared to an isocaloric product (*p* = 0.02). The GRV at the morning and evening measurements showed no circadian rhythm. When comparing the first and last ultrasound examination of each patient, there was a tendency towards an increased GRV (*p* = 0.07). Conclusion: The GRV measured by miniaturized ultrasound devices can provide important information about ICU patients without restricting treatment procedures in the ICU. Measurements are possible while EN therapy is ongoing. Further studies are needed to establish gastric ultrasound as a management tool in nutrition therapy.

## 1. Introduction

Nutrition is essential for recovery and vitality as each body requires an individual amount of carbohydrates, protein, fat, vitamins, and minerals for physiological functioning [1]. However, many patients are restricted in their dietary intake for various reasons. This is relevant for critically ill patients in particular. In the context of intensive care treatment, especially in cases of reduced nutritional intake and intestinal dysfunction, enteral nutrition plays an important role [2]. In cases of existing or impending malnutrition, EN can be administered by gravity drainage or active suctioning. Liquid nutrition can be administered continuously or on an intermittent basis.

Early initiation of EN with adequate nutrient delivery improves clinical outcome and is considered a proactive therapeutic strategy to reduce complications [2,3,4].

Like any medical procedure, EN is associated with risks. A frequent complication is regurgitation of gastric contents into the bronchial system. This can result in aspiration pneumonia, which leads to acute respiratory failure in up to 30% of patients in ICUs and death in 16% of cases [5].

GRV is taken to assess the risk of aspiration, nutrient tolerance, and gastric emptying [6] of patients in ICUs. Several methods are available for this purpose. The reflux test works by gravity drainage into a reservoir and is used in daily routine [7,8]. However, the test is controversial due to standardization [7,9]. Therefore, the determination of the GRV in the routine care of enterally fed intensive care patients is no longer generally recommended since the current joint guidelines of the Society of Critical Care Medicine (SCCM) and the American Society for Parenteral and Enteral Nutrition (ASPEN) [3].

Gastric-emptying disorders may occur frequently in patients requiring intensive care [9]. Possible complications such as aspiration during nutrition therapy to critically ill patients could be prevented by monitoring the GRV [10]. For this reason, there is a need for a method that can reliably and practicably monitor GRV.

US devices are widely available in ICUs and offer a non-invasive way to determine the GRV. The handling is familiar to most clinicians, and gastric sonography can be learned with a manageable amount of time [11].

In this regard, gastric sonography can provide quantitative and qualitative information about the gastric contents [12], and thus offers more advantages than the reflux test, which only allows for a quantitative determination of the gastric contents.

In 2009, Perlas et al. established a formula for the standardized determination of GRV by measuring the gastric antrum in patients in the right lateral decubitus position [13]. As measurement in this position is not always possible in intensive care patients, a formula was developed by Bouvet et al. in 2011 that allows for the determination of the GRV in the supine position [14].

The measurements are also possible with miniaturized US devices, so-called “pocket devices” [15,16]. Despite this, no studies have been performed on ultrasound-assisted monitoring of GRV in the context of EN in a close-meshed longitudinal setting.

Therefore, the aim of this study was to determine the GRV in the longitudinal course in critically ill patients using a pocket device in order to gain insight into how GRV behaves over time during the ICU stay.

## 2. Materials and Methods

After obtaining local ethics committee approval (State Chamber of Physicians of Saarland No. 57/19), all patients of the interdisciplinary surgical ICU of the Saarland University Medical Center who were fed enterally during the study period from June to December 2019 were evaluated for inclusion in the study. Exclusion criteria comprised age younger than 18, preceding surgical gastrectomy, prone position, pregnancy, and patients with abdominal vacuum therapy, if the wound sponge restricted the access to the epigastrium necessary for the US investigation. Written informed consent was obtained from all patients involved in the study or their legal representatives.

All patients were examined with the pocket device iViz (P20018-03, FUJIFILM Sonosite, Inc., Bothell, WA, USA) with a touchscreen. For all examinations the convex transducer model C60v (2–5 MHz, scan depth: 30 cm) was used and the standard setting “Abdomen” without image optimization.

The measurements were started at the next possible date after written informed consent. Patients were examined twice daily until the EN was terminated, relocated, or until the patient’s death. Usually, the 1st measurement was performed in the morning between 6:00 a.m. and 10:00 a.m., and the 2nd measurement in the afternoon/evening between 4:00 p.m. and 8:00 p.m. without a passive reflux test in the period of 2 h before the measurement. Occasionally, this period had to be deviated from or measurements had to be omitted in order not to influence clinical procedures, e.g., due to operative interventions or examinations. All examinations in this study were performed by the first author himself, so that variations in the measurements due to the examiner could be excluded. To obtain comparable results, all measurements were performed in a supine position.

Prior to each ultrasound examination, the bowel sounds were auscultated and grouped into three categories (sparse/normal/excited).

The average values were obtained from the three measurements per study, and the cross-sectional area was calculated according to Perlas et al. [13] using AP and CC distances and the GRV according to Bouvet et al. [14]. For better comparability, the volumes were also calculated in mL/kg body weight (bw). The dependent two-sample t-test was used to compare the study results. Two-sided *p*-values < 0.05 were regarded as significant. Demographic data and data on hospitalization and health status were included. The APACHE II, SAPS II, and SOFA scores were documented at patient admission and because of the partial delay in starting enteral nutrition before the first measurement. Patient-related data were collected from the hospital information system (i.s.h.med of SAP SE, Berlin, Germany) and the patient data management system of the interdisciplinary operative ICU (COPRA PDMS of COPRA System GmbH, Berlin, Germany). All data were anonymized.

## 3. Results

### 3.1. Patient Collective and Clinical History

The study included 18 critically ill patients who required intensive medical therapy in the interdisciplinary surgical ICU. Patients were examined twice daily as long as the medical indication for EN via nasogastric tube existed. A total of 217 examinations, and thus 651 single measurements, were performed and evaluated. The demographics are given in Table 1.

The average length of stay of patients in the interdisciplinary surgical ICU was 29.0 ± 19.3 days; and three patients (16.7%) died during their stay.

Patients were mechanically ventilated in 48.4% of the examinations (*n* = 105). In 29 cases (13.4%), patients were treated with catecholamines on examination.

The summary of APACHE II, SAPS II, and SOFA data are given in Table 2.

### 3.2. Nutrition

In total, 163 examinations (75.1%) took place during ongoing nutritional therapy. In the remaining 54 examinations (24.9%), nutritional intake was interrupted during the measurement. In addition to the standard preparations of Fresubin^®^ original fibre and Fresubin^®^ energy fiber, the patients were fed with the products Fresubin^®^ 2 kcal HP and Fresubin^®^ HP energy. The characteristics and frequency of use of the nutritional products at the time of the study are shown in Table A1.

Furthermore, the pump rate of EN at the time of the examination, the average pump rate, and the cumulative total amount of food supplied between the current and the last US examination were calculated (see Table A2). The average time between measurements was 13.8 ± 9.8 h.

In 1 case (0.5%), vomiting was documented in the time between two examinations; in 27 cases (12.4%), the patients took in oral food in addition to EN. In most cases (*n* = 25), this involved liquid foods, and in two cases it involved solid foods.

### 3.3. Ultrasound Examinations

#### 3.3.1. Feasibility of the Measurements

The first finding is that performance of the US examination was possible in all 217 cases with three measurements each, irrespective of pre-existing diseases, the diet, or the condition of the patients or any clinical parameters. Additionally, no medical examinations or nursing activities were delayed during this observational study. Moreover, the patients did not have to be prepared for the measurements, for example by adjusting the EN, the ventilation, or the medication.

#### 3.3.2. Results of the Ultrasound Examinations

**Figure 1:**
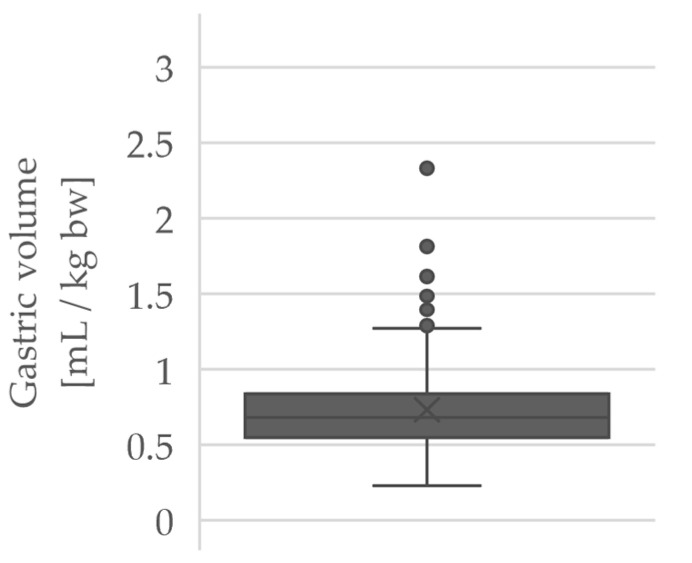
Measurement results of GRV, calculated according to Bouvet et al. (formula for measurements in supine position) [14] in mL/kg bw.

Figure 1 shows the measured GRV as a box plot.

The average CSA was 9.9 ± 3.6 cm^2^. The GRV was calculated to be 67.4 ± 21.3 mL or 0.73 ± 0.30 mL/kg bw.

**Figure 2:**
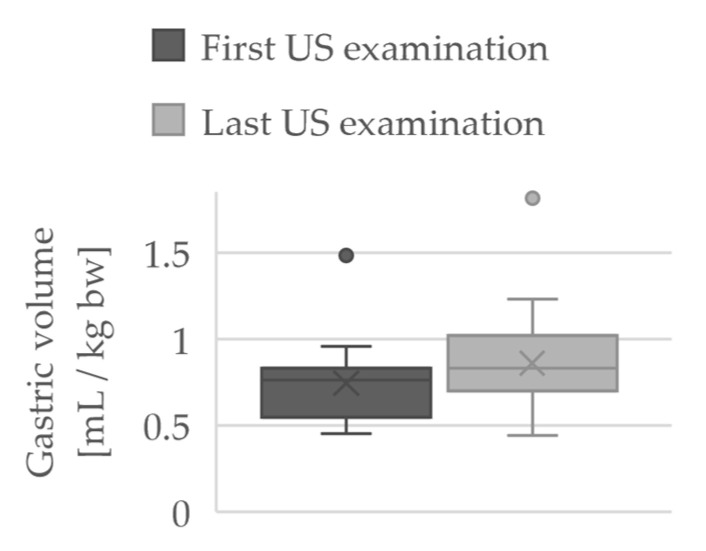
Boxplots of the GRV in mL/kg bw at first and last US examination.

In order to show the longitudinal course, the first US measurement of each patient was compared with the last measurement of the study performed with regard to the GRV in mL/kg bw. These results are shown in Figure 2.

Although the comparison did not show a significant difference (*p* = 0.07), the tendency is that the calculated GRV was larger in the last measurement (0.86 ± 0.33 mL/kg bw) than in the first examination (0.74 ± 0.25 mL/kg bw), regardless of the total number of examinations.

**Figure 3:**
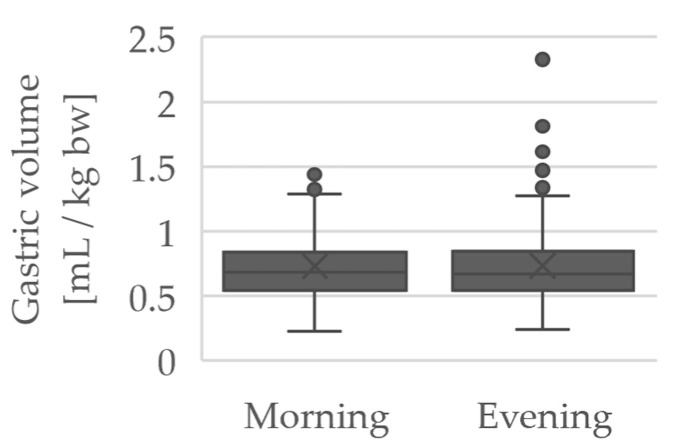
Boxplots of the GRV in mL/kg bw for the morning and evening measurements.

Furthermore, the measurement results of the morning (between 6:00 a.m. and 10:00 a.m.) and evening (between 4:00 p.m. and 8:00 p.m.) measurements were analyzed for a difference regarding the measured GRV. The mean values were almost identical (morning measurement: 0.73 ± 0.27 mL/kg bw, evening measurement: 0.73 ± 0.33 mL/kg bw), as shown in Figure 3.

### 3.4. Auscultation and Digestion Findings

Normal bowel sounds were auscultated in 51.2% of the examinations (*n* = 111), sparse bowel sounds were detected in 13.8% of the examinations (*n* = 30), and excited bowel sounds in 35.0% of the examinations (*n* = 76).

The patients’ defecations were documented in the period between the current measurement and the last examination. In 44.7% of the examinations (*n* = 97), the patient did not have a stool since the last measurement. Solid stool was documented in 0.9% (*n* = 2) and pulpy stool in 12.0% (*n* = 26) of cases. Liquid-pulpy (6.5%, *n* = 14) and liquid (35.0%, *n* = 76) stools were the most common overall. Swish enemas were performed in two cases (0.9%).

### 3.5. Comparison of the Measurement Results with the Dietary Pattern

The GRV in mL/kg bw was considered in relation to the pump rate of EN. For this purpose, the pump rate was grouped in 20 mL/h intervals. On comparison, it became clear that the mean values of the individual groups were similar (<20 mL/h: 0.70 ± 0.25 mL/kg bw; 20–39 mL/h: 0.71 ± 0.43 mL/kg bw; 40–59 mL/h: 0.65 ± 0.15 mL/kg bw). Considering only different pump rates, no effect on the GRV was observed.

**Figure 4:**
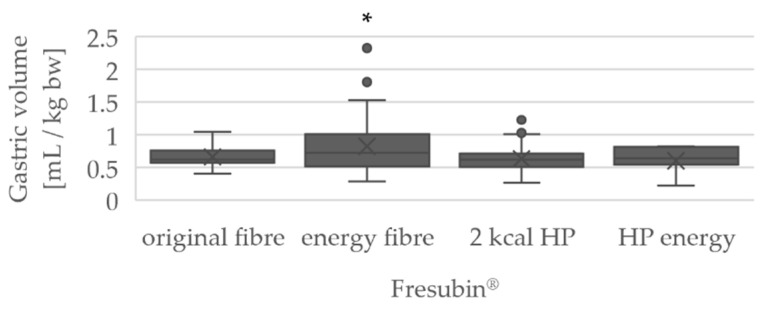
Boxplots of the GRV in mL/kg bw according to nutritional product used, * *p* < 0.05 (Fresubin^®^ energy fiber versus original fiber). For the quantity of uses of the products, see Table A1.

The measurement results for the GRV in mL/kg bw were also grouped according to the nutritional product used, as shown in Figure 4.

If the patient was fed with the standard product Fresubin^®^ original fiber, the average GRV was 0.66 ± 0.15 mL/kg bw. With the second standard product Fresubin^®^ energy fiber, an average GRV of 0.83 ± 0.40 mL/kg bw was measured. When both standard products were compared with each other, the GRV was significantly greater when the high-calorie nutritional preparation Fresubin^®^ energy fiber was used (*p* = 0.02). The two other products, Fresubin^®^ 2 kcal HP and Fresubin^®^ HP energy, showed a similar mean value with regard to the GRV (2 kcal HP: 0.64 ± 0.22 mL/kg bw; HP energy: 0.61 ± 0.20 mL/kg bw).

### 3.6. Comparison of the Measurement Results with the Bowel Sounds

**Figure 5:**
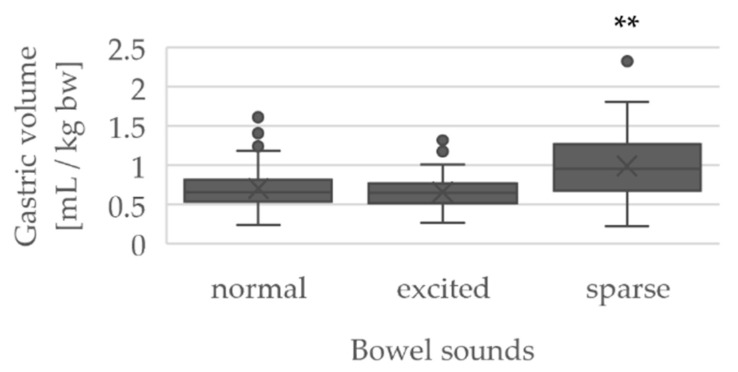
Boxplots of the GRV in mL/kg bw according to auscultation findings of bowel sounds, ** *p* < 0.01 (sparse bowel sounds versus normal and excited bowel sounds), normal *n* = 111, excited *n* = 76, and sparse *n* = 30.

The grouping of the GRV in mL/kg bw according to bowel sounds (normal/excited/sparse) is shown by boxplots in Figure 5.

The GRV was similar in normal (0.71 ± 0.26 mL/kg bw) and excited (0.66 ± 0.22 mL/kg bw) bowel sounds. However, the GRV was significantly increased in sparse (0.99 ± 0.43) bowel sounds compared to normal (*p* < 0.01) and excited bowel sounds (*p* < 0.01).

## 4. Discussion

To the best of our knowledge, this is the first study of ultrasound-assisted monitoring of GRV during EN in a close longitudinal follow-up. The main finding is that the monitoring of GRV in ICU patients is feasible using a pocket device. Neither EN nor clinical treatment processes had to be interrupted in the process.

### 4.1. Assessment of GRV

The monitoring of GRV in ICU patients is no longer recommended in the current joint low-evidence guidelines of SCCM and ASPEN [3]. The most important reason for this is that EN must be interrupted to determine the GRV. Since EN is administered continuously in most cases, a significant amount of infused nutritional product is missing due to the interruption time [3,17].

However, the amount of nutrition administered is related directly to the prognosis of the disease and the course of the ICU stay. If EN does not have to be stopped because of the routine monitoring of GRV, the daily prescribed nutrient requirement can be managed better [17,18,19].

The significance of measuring GRV in ICUs is controversial. A clear significance to reduce aspiration pneumonia has not been demonstrated in some studies [17,20,21,22,23], but a lower incidence of vomiting with lower GRV has [20,24]. Therefore, in its 2019 guideline, ESPEN considers it reasonable to continue to use GRV to detect intolerance to EN [10]. If the GRV threshold at which nutritional therapy is switched or discontinued or set too low, the patient may receive too few nutrients even though there is no increased risk of aspiration [25].

Consideration must therefore be given to whether interruption of EN justifies the control of GRV. Furthermore, it has to be considered that, in the case of increased GRV, the reduction or discontinuation of EN on the one hand represents a risk in terms of reduced nutrient intake, but on the other hand also offers protection against vomiting.

Gastric ultrasound is a way to determine the GRV without having to interrupt EN, as our study was able to show. Therefore, at least the failure of the infused food volume during the examination can be avoided [26].

Thus, when checking the GRV with ultrasound, vomiting can be probably prevented at the same time and nutrient intake can be better achieved.

### 4.2. Use of Pocket Devices

In comparison to standard US devices, pocket devices have a smaller size and weight, may bring time savings [27], can be used more easily in the hospital setting, and are also available for emergency medicine or home visits. These attributes could be confirmed in our study, especially due to the small size of the device and the fast set-up and take-down, the examinations could be well integrated into the clinical routine. The US examination was possible in 100% of the cases. In particular, the patients did not have to be prepared for the measurements. As a result, high clinical reproducibility can be assumed.

Gastric US with pocket devices has been presented in two studies to date. Kaydu and Gokceket (2018) studied the GRV in 120 patients in relation to the duration of food abstinence before anesthetic induction [16]. With increasing duration of food abstinence, the CSA decreased significantly. Fasting or malnutrition is therefore also visible based on the GRV using ultrasound. Skornik (2018) tested the feasibility of sonography-assisted measurement of the GRV of enterally fed ICU patients with the pocket device V-Scan, comparing the results with the standard device Vivid i9 (both GE Healthcare) and the passive reflux test [15]. The measurement results with the pocket device correlated strongly with those of the standard device and the positive passive reflux test. In this regard, the results are comparable to current methods for monitoring GRV.

### 4.3. Measurement Results Analysis

#### 4.3.1. GRV Compared to Bowel Sounds

When comparing auscultated bowel sounds with the measured GRV in this study, the results were similar for normal and excited bowel sounds. In sparse bowel sounds, however, the measured GRV seems significantly increased compared to normal and exited bowel sounds.

Although auscultation of bowel sounds is considered an integral part of the clinical examination, its diagnostic significance is widely unexplored [28]. Nevertheless, in some cases, auscultation may provide evidence of pathology. In a review paper, Li et al. presented the relationships between bowel sounds and gastrointestinal motility in critically ill patients [29].

However, there is no standard terminology for the classification of bowel sounds [30], which limits comparability with other studies. Taken together, we believe that gastric ultrasound could be a solution for an objectifiable assessment of GI motility through the association with bowel sounds demonstrated in this study.

#### 4.3.2. GRV at Morning and Evening Measurements and at First and Last Ultrasound Examination

When comparing the morning and evening examinations, the mean values of the measurement results were approximately constant, so that no circadian variation of the GRV was detected in this study.

In a physiological setting, the GI tract is subject to circadian rhythms that coordinate peripheral organs and control, among other things, enzyme production, which adjusts the body to alternating phases of food intake and fasting [31,32]. This can be severely affected in critically ill patients [9,17,33].

Therefore, gastric ultrasound in ICUs may play an important role in future studies to improve our knowledge of the circadian rhythms of ICU patients with respect to gastrointestinal function.

Furthermore, there was a tendency for the last measurement to indicate a larger GRV than the initial measurement. A stay of at least 24 h in the ICU is associated with the development of gastrointestinal dysfunction [34,35], as evidenced by, among other things, decreased GI motility [36], which causes increased GRV [17]. The small number of patients in this study as well as the different length of stay in the ICU might be the reason why no significant results could be shown.

#### 4.3.3. GRV by Using Different Nutritional Products

In our study, a total of four nutritional products were recorded. The measured GRVs were similar for the products Fresubin^®^ original fiber, Fresubin^®^ 2 kcal HP, and Fresubin^®^ HP energy. Only in the case of the high-calorie product Fresubin^®^ energy fiber were larger volumes measured. In addition to a higher number of calories, this product also contains less dietary fiber.

Additionally, other studies investigated the effects of different nutritional products on the GI tract of ICU patients. Spindler-Vesel et al. showed a larger GRV in enterally fed ICU patients on high-fiber standard diets than on low-fiber, high-calorie diets [37]. Chittawatanarat et al. also investigated the effects of dietary products on digestion [38]. In contrast, Yagmurdur et al. found no significant differences in the GRV between cohorts on high-fiber and low-fiber diets [39].

In conclusion, the study situation regarding the influence of different dietary products on the GRV in ICU patients is controversial. Even major reviews do not reach a clear conclusion [40,41], which might be related to the dynamics of the metabolism of critically ill patients as well as the consequent non-constant energy demand [41]. It is necessary to conduct further studies to make clear recommendations on nutrient intake for specific patient groups.

### 4.4. Limitations

The patient population contained 83.3% (*n* = 15) male patients and 16.7% (*n* = 3) female patients. The patient collective also had an inhomogeneous structure in terms of duration of hospitalization, number and type of surgery, and medication. The aim of the work was to show the longitudinal course of the patients who were fed enterally. For this purpose, all enterally fed critically ill patients were included, with the exception of the above-mentioned patient groups. Therefore, it was more important to represent a cross-section of the patient collective of the local ICU than a homogeneous, selected patient group for better reproducibility. Results must be interpreted carefully due to low patient numbers. The individual examinations were not independent, as they were performed on the same patient cohort. The possibilities for statistical analyses were therefore limited.

### 4.5. Conclusions

The GRV measured by miniaturized ultrasound can provide important information about ICU patients without restricting treatment procedures in the ICU. The measurements are possible during ongoing EN therapy. The examinations can be performed intuitively after brief training, and the procedure provides more extensive information beyond auscultation and passive reflux testing.

In the future, gastric ultrasound could play an important role in planning enteral feeding therapy. Adjustment of the pump rate would be possible under ultrasound guidance; at the same time, complications such as aspiration could be reduced.

Further studies with higher patient numbers are needed to confirm the results of this study and to establish gastric ultrasound in feeding therapy.

## Figures and Tables

**Table 1:** Demographics.

Characteristics	Frequency or Mean ± SD
Gender (Male)	15 (83.3%)
Age (years)	66.9 ± 10.4
Height (cm)	176 ± 8
Body weight (kg)	88 ± 15
BMI (kg/m^2^)	29 ± 4
Coronary heart disease	4 (22.2%)
Diabetes mellitus 2	2 (11.1%)
Stroke	5 (27.8%)
Trauma surgery	6 (33.3)
Abdominal surgery	7 (38.9%)
Other (surgery)	5 (27.8%)

**Table 2:** Scores of patient condition and clinical progress.

	On Arrival (ICU)	Before First Measurement
APACHE II	19.7 ± 3.3	16.4 ± 4.5
SAPS II	38.1 ± 7.5	32.2 ± 10.9
SOFA	4.8 ± 1.7	3.6 ± 2.9

APACHE II: Acute Physiology and Chronic Health Evaluation, SAPS II: Simplified Acute Physiology Score, SOFA: Sequential Organ Failure Assessment Score.

## Data Availability

The data presented in this study are available on request from the corresponding author. The data are not publicly available due to privacy protection.

## Appendix A

**Table A1:** Characteristics [42] and use of nutritional products at the time of study.

Product: Fresubin^®^	Contents	Use at the Time of Examination
original fiber (isocaloric, with dietary fiber, low protein)	Calories: 1.0 kcal/mL	32 (19.6%)
	Dietary fiber: 1.5 g/100 mL	
	Protein: 3.8 g/100 mL	
energy fiber (high caloric, with dietary fiber, low protein)	Calories: 1.5 kcal/mL	48 (29.4%)
	Dietary fiber: 1.1 g/100 mL	
	Protein: 3.8 g/100 mL	
2 kcal HP (high caloric, without dietary fiber, high in protein)	Calories: 2.0 kcal/mL	72 (44.2%)
	Dietary fiber: 0.0 g/100 mL	
	Protein: 10.0 g/100 mL	
HP energy (high caloric, without dietary fiber, high in protein)	Calories: 1.5 kcal/mL	11 (6.7%)
	Dietary fiber: 0.0 g/100 mL	
	Protein: 7.5 g/100 mL	
Total		163 (100.0%)

**Table A2:** Run rate of EN at the time of examination, average run rate since the last examination, and cumulative total since the last examination.

Product: Fresubin^®^	Run Rate at the Time of Examination	Average Run Rate Since the Last Examination	Cumulative Total Since the Last Examination
original fiber	41.4 mL/h ±22.1 mL/h	38.5 mL/h ±19.1 mL/h	507.5 mL ±336.8 mL
energy fiber	34.6 mL/h ±17.3 mL/h	34.9 mL/h ±13.6 mL /h	421.0 mL ±204.8 mL
2 kcal HP	33.4 mL/h ±13.8 mL/h	32.9 mL/h ±9.8 mL/h	463.5 mL ±413.3 mL
HP energy	42.0 mL/h ±0.0 mL/h	39.3 mL/h ±3.7 mL/h	461.1 mL ±64.6 mL
Total	36.0 mL/h ±16.9 mL/h	35.1 mL/h ±13.4 mL/h	460.0 mL ±334.0 mL
